# CXCL6‐EGFR‐induced Kupffer cells secrete TGF‐β1 promoting hepatic stellate cell activation via the SMAD2/BRD4/C‐MYC/EZH2 pathway in liver fibrosis

**DOI:** 10.1111/jcmm.13787

**Published:** 2018-08-14

**Authors:** Xiaobo Cai, Zhenghong Li, Qidi Zhang, Yin Qu, Mingyi Xu, Xinjian Wan, Lungen Lu

**Affiliations:** ^1^ Department of Gastroenterology Shanghai General Hospital Shanghai Jiao Tong University School of Medicine Shanghai China

**Keywords:** CXCL6, hepatic stellate cells, Kupffer cells, liver fibrosis

## Abstract

Liver fibrosis is the excessive accumulation of extracellular matrix proteins in response to the inflammatory response that accompanies tissue injury, which at an advanced stage can lead to cirrhosis and even liver failure. This study investigated the role of the CXC chemokine CXCL6 (GCP‐2) in liver fibrosis. The expression of CXCL6 was found to be elevated in the serum and liver tissue of high stage liver fibrosis patients. Furthermore, treatment with CXCL6 (100 ng/mL) stimulated the phosphorylation of EGFR and the expression of TGF‐β in cultured Kupffer cells (KCs). Although treatment with CXCL6 directly did not activate the hepatic stellate cell (HSC) line, HSC‐T6, HSCs cultured with media taken from KCs treated with CXCL6 or TGF‐β showed increased expression of α‐SMA, a marker of HSC activation. CXCL6 was shown to function via the SMAD2/BRD4/C‐MYC/EZH2 pathway by enhancing the SMAD3‐BRD4 interaction and promoting direct binding of BRD4 to the *C‐MYC* promoter and CMY‐C to the *EZH2* promoter, thereby inducing profibrogenic gene expression in HSCs, leading to activation and transdifferentiation into fibrogenic myofibroblasts. These findings were confirmed in a mouse model of CCl_4_‐induced chronic liver injury and fibrosis in which the levels of CXCL6 and TGF‐β in serum and the expression of α‐SMA, SMAD3, BRD4, C‐MYC, and EZH2 in liver tissue were increased. Taken together, our results reveal that CXCL6 plays an important role in liver fibrosis through stimulating the release of TGF‐β by KCs and thereby activating HSCs.

## 1 INTRODUCTION

Liver fibrosis is the excessive accumulation of extracellular matrix (ECM) proteins, including collagen and fibronectin, in response to the persistent inflammation and cell death that occurs during the pathogenesis of most types of chronic liver disease. This excessive deposition of ECM proteins at sites of tissue damage, resulting from, for example, viral infection or alcoholic liver disease, is a consequence of a dysregulated wound‐healing response. Although reversible if the source of the injury is eliminated, unchecked liver fibrosis can develop into cirrhosis and even liver failure due to portal hypertension, with the only treatment option being transplantation.^1^, ^2^ Chronic liver disease is a major cause of mortality worldwide and correlates with an increased risk of hepatocellular carcinoma.^3^

Hepatic stellate cells (HSCs), which are characterized by the storage of retinyl esters in cytoplasmic lipid droplets, constitute around 10% of resident liver cells and in healthy livers are phenotypically non‐proliferative and quiescent. However, HSCs are the predominant precursor cells of liver myofibroblasts and upon activation, which is triggered by signalling pathways such as those involving platelet‐derived growth factor (PDGF) and transforming growth factor‐beta (TGF‐β), dramatic phenotypic changes are induced accompanied by transdifferentiation into proliferative, inflammatory, fibrogenic myofibroblasts. This process of HSC activation and transdifferentiation is the driver of fibrosis in liver injury.^4^ However, the pathways and mediators involved in HSC activation are complex. Understanding the complex regulation of HSC activation is crucial for the development of novel therapeutic strategies to treat liver disease.

TGF‐β, which is secreted by several cell types in the liver, is considered the predominant cytokine to trigger HSC activation and fibrogenic transdifferentiation.^5^, ^6^ Kupffer cells (or stellate macrophages) are specialized macrophages that reside in the liver and release proinflammatory and profibrogenic factors such as TGF‐β in response to various stimuli (including CXC chemokines), that in turn modulate HSC behaviour and trigger HSC activation.^7^ TGF‐β‐induced de novo synthesis of alpha smooth muscle cell actin (αSMA) fibres enhances the contractility of the cells and increases the expression of ECM proteins for ECM remodelling.^8^ TGF‐β also binds and phosphorylates the type I receptor, which in turn induces phosphorylation of downstream SMAD proteins, which translocate to the nucleus and activate various mitogen−activated protein kinase (MAPK) signalling pathways to trigger HSC activation and myofibroblast transformation, such as the extracellular signal−regulated kinase (ERK), p38 and c−jun N−terminal kinase (JNK) pathways.^9^, ^10^, ^11^, ^12^, ^13^

Previous studies reported that SMAD proteins can bind to BRD4 in response to TGF‐β stimulation.^14^ BRD4 is a global regulator of enhancer−mediated profibrogenic gene expression in HSCs.^15^ There is evidence from various cancer cell types that BRD4 can interact with the *C‐MYC* promoter and directly regulate its transcriptional expression.^16^, ^17^, ^18^ Furthermore, recent studies by one research group into the role of BRD4 in bladder cancer reported that BRD4 positively regulates enhancer of zeste homologue 2 (*EZH2*) transcription through the upregulation of the *C‐MYC* promoter.^19^, ^20^

In this study, the role of CXCL6 (GCP‐2) in liver fibrosis was investigated. The subfamily of CXC chemokines that possess an ELR motif are potent neutrophil chemoattractants and interact with the G protein‐coupled receptors, CXCR1 and/or CXCR2.^21^ Among this subfamily, CXCL6 has been shown to play a role in neutrophil recruitment leading to tissue damage and prolonged inflammatory responses.^22^ CXCL6 has thereby been proposed to contribute to fibrosis and CXC chemokines have been proposed as prognostic biomarkers of liver fibrosis.^23^ Our findings revealed a correlation between elevated CXCL6 levels in serum and liver tissues and high stage liver fibrotic disease in patients. By employing in vitro experiments and a carbon tetrachloride (CCl_4_)‐induced fibrosis mouse model,^24^ CXCL6 was shown to promote the release of TGF‐β by Kupffer cells (KCs), leading to HSC activation. Our findings provide important insight into the complex mechanisms of HSC activation that contribute to liver fibrosis.

## 2 MATERIALS AND METHODS

### 2.1 Human serum and liver samples

Serum samples were taken from 50 patients with clinically diagnosed liver fibrosis who had been classified according to fibrotic staging (S) (n = 10 samples for each of the stages: S0, S1, S2, S3 and S4). Liver tissues were taken from 10 patients with clinically diagnosed liver hepatitis who had been classified according to fibrotic staging (S) (n = 6 samples from each of the stages: S0, S1, S2 and S4). All patients were admitted to our hospital from 2013 to 2015. Ethical approval for the study was provided by the independent ethics committee of Shanghai General Hospital, affiliated with Shanghai Jiao Tong University School of Medicine. Informed and written consent were obtained from all patients or their advisors according to the ethics committee guidelines.

### 2.2 Liver histological observations

Slices of human liver were fixed in 10% phosphate‐buffered saline (PBS)‐formalin for at least 24 hour and then embedded in paraffin for histological assessment of tissue damage. Samples were subsequently sectioned (5 μm), stained with haematoxylin and eosin (H&E) using standard protocols and then examined microscopically under a light microscope (Olympus Corporation, Tokyo, Japan) to evaluate structural changes indicating liver damage.

### 2.3 Immunohistochemistry

Liver tissue sections were initially treated by deparaffinization and hydration. Then EDTA (pH 8.0) was added and antigen retrieval was performed by heating at 100°C for 5 minutes in 10 mm citrate buffer. The slide‐mounted sections were then incubated with CXCL6 antibody (1:500, Santa Cruz Biotechnology, Santa Cruz, CA, USA) for 1 hour at room temperature, followed by incubation with biotin‐labelled secondary antibodies. Immunohistochemical signals were detected by treatment with 3,3‐diaminobenzidine (DAB; Shanghai Long Island, Co., Ltd., China) solution and counterstaining with hematoxylin (BASO, China), followed by microscopic evaluation of positively stained cells (Olympus Corporation).

### 2.4 Biochemical analysis

ALT, AST, and hydroxyproline levels were analysed using commercial kits according to the manufacturers’ protocols (Nanjing Jiancheng Bioengineering Institute).

### 2.5 Cell culture

Hepatic stellate cell‐T6 cells (HSCs) were purchased from the Cell Bank at Chinese Academy of Sciences (Shanghai, China) and the isolation of primary KCs and HSCs from rats was performed as previously described.^25^, ^26^ Cells were cultured in Dulbecco's modified Eagle's medium (DMEM; HyClone, Logan, UT, USA) supplemented with penicillin (100 IU/mL), streptomycin (100 mg/mL) and 10% (vol/vol) heat‐inactivated foetal bovine serum (FBS; Gibco, Carlsbad, CA, USA) at 37°C in a 5% CO_2_ incubator. Hepatic stellate cells were cultured for 48 hour and serum‐starved with 0.5% FBS for 24 hour before the experiments. CXCL6 and TGF‐β were purchased from R&D Systems (Minneapolis, MN, USA). Inhibitors (SCH‐527123, Afatinib, SB431542, JQ1, 10058‐F4 and EPZ005687) were purchased from Active Biochem (Maplewood, NJ, USA) and 1D11 was purchased from GeneTex (Irvine, CA, USA).

### 2.6 Experimental animals and the generation of a liver fibrosis mouse model

Male wild‐type C57BL/6 mice (16‐20 g) provided by Shanghai Laboratory Animal Center of Chinese Academy of Science (Shanghai, China) were used for all of the animal experiments. The animal room was maintained at a temperature of 22 ± 1°C with a 12 hour light‐dark cycle (6:00‐18:00) and 65 ± 5% humidity. All animals received humane care in compliance with the institutional animal care guidelines approved by the Experimental Animal Ethical Committee of Shanghai Jiao Tong University.

For the experiments, 50 mice were randomly divided into five groups (n = 10). Mice received a single intraperitoneal (i.p.) injection of CCl_4_, 0.6 μL/g body weight, diluted 1:3 in corn oil (Sigma‐Aldrich, St. Louis, MO, USA), twice per week. The control group received a single i.p. injection of corn oil only and were euthanized after 8 weeks. The experimental groups were euthanized and plasma and liver tissue were collected after 2, 4, 6 and 8 weeks.

### 2.7 RNA isolation and quantitative real‐time PCR

RNA was isolated using TRIzol reagent (Invitrogen, Carlsbad, CA, USA) according to the manufacturer's instructions and reverse transcribed using the miScript Reverse Transcription kit (Qiagen, Hilden, Germany). QRT‐PCR was performed using the SYBR Premium Ex Taq II kit (Takara, Dalian, China) in an ABI PRISM 7500 Sequence Detection System (Applied Biosystems, Foster City, CA, USA). All reactions were performed in triplicate and the mean values obtained were used to calculate expression levels after normalization to β‐actin, which was used as an internal standard. The primer sequences used in this study are listed in Table 1.

**Table 1:** Sequences of primers for quantitative real‐time PCR

	Gene name	Forward primer sequence (5′ → 3′)	Reverse primer sequence (5′ → 3′)
Mouse	α‐SMA	CCTTCGTGACTACTGCCGAG	GTTTCGTGGATGCCCGCTG
	TGF‐β1	TGGCCAGATCCTGTCCAAAC	CATAGATGGCGTTGTTGCGG
	BRD4	TGCTCAGAGTGGTGCTCAAG	CCGTGGATACACCAGGCTTT
	C‐MYC	TCCATCCTATGTTGCGGTCG	CGCTCCACATACAGTCCTGG
	EZH2	GTGCTCTGCCTCCTGAATGT	GTGCTGGGTCTGCTACTGTT
	β‐actin	TTCGTTGCCGGTCCACACCC	GCTTTGCACATGCCGGAGCC
Rat	α‐SMA	CATCCGACCTTGCTAACGGA	AGTCCAGAGCGACATAGCAC
	TGF‐β1	CACTGATACGCCTGAGTGGC	GGAAGGGTCGGTTCATGTCA
	BRD4	TGAGCCTGAAGAGCCAGTTG	CACATTGCTGTTGCTGCTGT
	C‐MYC	CAGCTCGCCCAAATCCTGTA	GCCTCTTGATGGGGATGACC
	EZH2	CCAGATAAGGGCACAGCAGAA	GGGTGTTGCATGAAAGGGATG
	β‐actin	GAACCCTAAGGCCAACCGTG	AACCGCTCATTGCCGATAGT

### 2.8 Enzyme‐linked immunosorbent assay (ELISA)

The levels of CXCL6, α−smooth muscle actin (α‐SMA) and TGF‐β in sera or cell culture supernatants were determined using an ELISA kit (Nanjing Jiancheng Bioengineering Institute) according to standard protocols.

### 2.9 Immunofluorescent staining

Hepatic stellate cells were fixed in 4% paraformaldehyde solution (pH 7.4), blocked in 5% bovine serum albumin (BSA) or 1% BSA with normal serum in 0.1% Triton X‐100, and then incubated with primary/secondary antibodies or rhodamine‐phalloidin (Cytoskeleton, Denver, CO, USA). The cells were then counterstained with 4ʹ, 6‐diamidino‐2‐phenylindole (DAPI). Images were obtained by fluorescence microscopy at a magnification of 200 × , or using an LSM 510 META laser confocal microscopy system (Carl Zeiss, Jena, Germany).

### 2.10 Protein isolation and western blot analysis

Hepatic stellate cells or KCs were washed three times with PBS, harvested, and lysed in co‐immunoprecipitation (co‐IP) buffer, as previously described.^27^ The total cell lysate (5 mg protein) was subjected to immunoprecipitation with the appropriate antibodies, as indicated, overnight at 4°C with gentle agitation, followed by incubation with protein A/G Plus‐agarose for 2 or 4 hour at 4°C. The immunocomplex was washed three times and then mixed with SDS sample buffer and boiled for 5 minutes. For western blotting, co‐precipitates or whole cell extracts were resolved by SDS‐PAGE and blotted on PVDF membranes (Millipore, Bedford, MA, USA). The membranes were immunoblotted with the indicated antibodies and developed using an ECL detection system (Applygen, Beijing, China). EGFR (phospho Y845) (1:1000); EGFR (1:1000); Erk1 (pT202/pY204) + Erk2 (pT185/pY187) (1:2000); ERK1 + ERK2 (1:2000); CXCR1 (1:2000); CXCR2 (1:2000); α‐SMA (1:2000); Smad3 (phospho S213) (1:1000); Smad3 (1:1000); BRD4 (1:2000); C‐MYC (1:2000); EZH2 (1:2000) and β‐actin (1:5000), were all purchased from Santa Cruz Biotechnology. The expression levels of proteins were determined using ImageJ software.

### 2.11 Chromatin immunoprecipitation (ChIP) assay

A ChIP assay was performed using an EZ‐ChIP kit (Upstate Biotechnology, Lake Placid, NY, USA), as previously described.^20^ Briefly, chromatin DNA was immunoprecipitated with the corresponding antibody or normal IgG. It was then washed and the DNA‐protein cross‐links were reversed. PCR primers to amplify the C‐MYC promotor (forward, 5ʹ‐ACATGCTATACACGCACCCC‐3ʹ and reverse, 5ʹ‐GCTCTCT GCCAGTCTGTACC‐3ʹ) and the EZH2 promoter (forward, 5ʹ‐TACCCCAACAGTTCATAGGTG‐3ʹ and reverse, 5ʹ‐GCAGTGCTTATCGCCCTAGA‐3ʹ) regions were designed using the Premier Primer 5.0 software and were used in PCR assays to assess the relative amount of immunoprecipitated DNA. A negative control was included to normalize the data.

### 2.12 Statistical analysis

Statistical analyses were performed using the SPSS 17.0 software (SPSS Inc., Chicago, IL, USA). Statistically significant differences were detected using either the Student's *t* test for comparison between the means or one‐way analysis of variance. Data are presented as the mean ± SD and are considered to be statistically significant at *P < 0.05, **P < 0.01, ***P < 0.001, ^#^ *P* < 0.05, ^##^ *P* < 0.01 and ^###^ *P* < 0.001.

## 3 RESULTS

### 3.1 Elevated expression of CXCL6 in the sera and liver tissue of liver fibrosis patients

**Figure 1:**
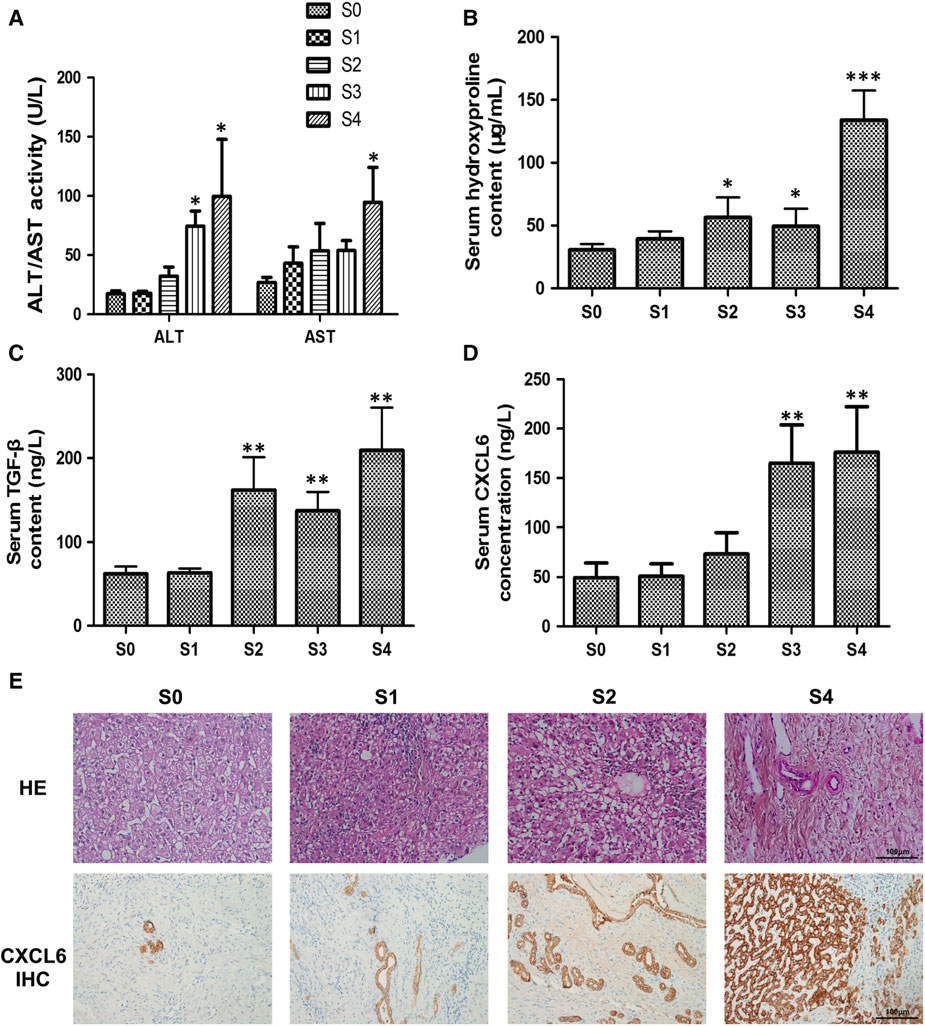
CXCL6 Expression is Higher in Liver Fibrosis Patients. A, ALT/AST activity of sera from patients at different fibrotic stages. B‐D, Levels of serum hydroxyproline, TGF‐β and CXCL6 content in patients at different fibrotic stages (n = 10). E, H&E staining of liver tissue. Typical images are chosen for each fibrotic stage (original magnification 200 × ). F, Immunohistochemical staining of CXCL6 of liver tissue (original magnification 200 × ) (n = 6). *P < 0.05, **P < 0.01, ***P < 0.001 vs S0 group

The sera of liver fibrosis patients categorized as belonging to different fibrotic stages (S1‐S4) were analysed biochemically using commercial kits to assess ALT/AST activity and hydroxyproline levels. The serum ALT/AST ratio is a commonly used biomarker of liver health, and hydroxyproline is a major constituent of collagen. Overall, a steady increase in ALT/AST activity and hydroxyproline levels accompanied the progression of liver fibrosis, with the highest levels evident in patients with fibrotic stage S4, as expected (Figure 1A, *P* < 0.05 for ALT and AST; Figure 1B, *P* < 0.001 for hydroxyproline vs the S0 group). Next, serum levels of TGF‐β and CXCL6 were determined for the various fibrotic stages. Again, an overall increase in TGF‐β and CXCL6 levels was detected with increased fibrotic staging, with the highest levels evident in patients with S4 fibrosis (Figure 1C, *P* < 0.01 for TGF‐β; Figure 1D, *P* < 0.01 for CXCL6 vs the S0 group). To confirm the increased expression of the chemokine CXCL6 in late‐stage fibrosis patients, immunohistochemical analysis was performed on liver sections. The increase in ECM deposition and cell death with advanced fibrotic staging evident by H&E staining of liver tissue (Figure 1E) correlated with increased expression of CXCL6, as demonstrated by immunohistochemical staining (Figure 1F).

### 3.2 CXCL6 stimulates TGF‐β secretion in Kupffer cells via the CXCR1/2‐EGFR pathway

**Figure 2:**
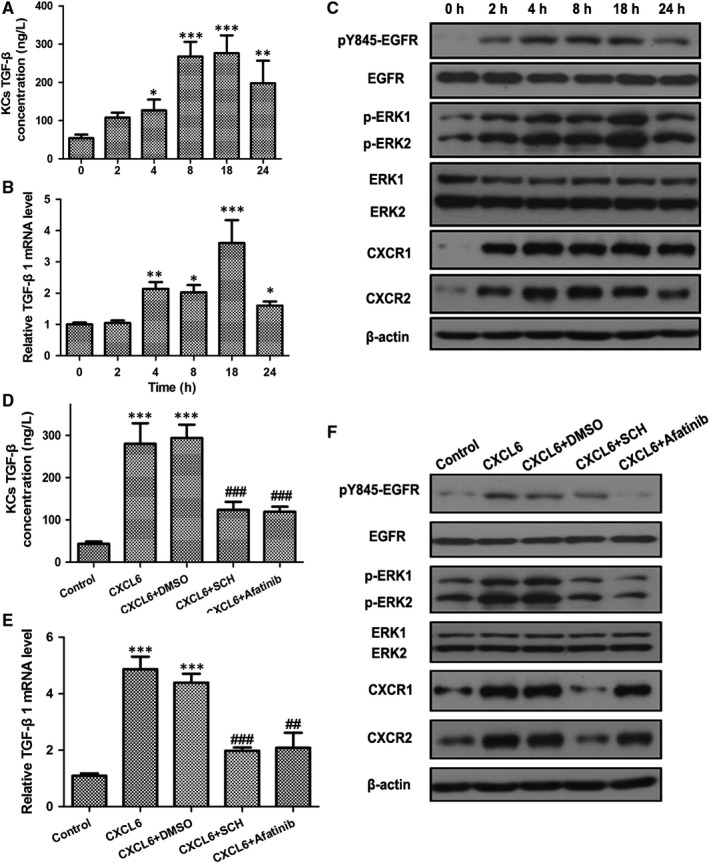
CXCL6 Stimulates TGF‐β Secretion in Kupffer Cells via the CXCR1/2‐EGFR Pathway. A and B, TGF‐β concentration and mRNA expression in Kupffer cells (KCs) incubated with CXCL6 (100 ng/mL) for the indicated times were detected by ELISA and RT‐PCR. C, KCs were incubated with CXCL6 (100 ng/mL) for the indicated times, and then cellular EGFR (phospho Y845), total‐EGFR, phospho‐ERK1/2, total‐ERK1/2, CXCR1 and CXCR2 protein levels were detected by western blot analysis. D and E, TGF‐β concentration and mRNA expression in KCs incubated with CXCL6 (100 ng/mL) or phosphate‐buffered saline (PBS) with the addition of CXCR1/2 antagonist SCH527123 (10 μmol/L), EGFR antagonist Afatinib (10 μmol/L), or DMSO as the control, for 18 h were detected by ELISA and RT‐PCR. E, Protein levels in KCs incubated with CXCL6 (100 ng/mL) or PBS with the addition of CXCR1/2 antagonist SCH527123 (10 μmol/L), EGFR antagonist Afatinib (10 μmol/L), or DMSO as a control, for 18 h were detected by western blotting (n = 3). *P < 0.05, **P < 0.01, ***P < 0.001 vs 0 h or control group, ^##^ *P* < 0.01, ^###^ *P* < 0.001 vs CXCL6 + DMSO group

Kupffer cells (KCs) are specialized macrophages that reside in the liver. To determine the effects of exposure to CXCL6, KCs were incubated with CXCL6 over a 24 hour time‐course and the TGF‐β concentration was determined by ELISA (Figure 2A). TGF‐β levels increased in the KCs up to 18 hour (*P* < 0.001 vs 0 hour), then decreased at 24 hour. This result was verified by RT‐PCR analysis of the mRNA expression levels of TGF‐β (Figure 2B). To investigate the pathway responsible for the CXCL6‐induced secretion of TGF‐β in KCs, CXCL6‐exposed KCs were subjected to western blot analysis of cellular EGFR (phospho Y845), total‐EGFR, phospho‐ERK1/2, total‐ERK1/2, CXCR1 and CXCR2 protein levels (Figure 2C). The phosphorylated forms of EGFR and ERK1/2 showed increased expression, peaking at 18 hour, which correlated with the kinetics of TGF‐β expression. Upregulated expression of CXCR1 and CXCR2 protein levels was also evident over the time‐course of CXCL6 exposure.

Next, to confirm the link between the CXCL6‐induced secretion of TGF‐β in KCs and CXCR1/2 and EGFR expression, ELISA was performed on KCs incubated with CXCL6 with or without the CXCR1/2 antagonist SCH527123 or the EGFR antagonist Afatinib for 18 hour (Figure 2D). Both SCH and Afatinib significantly attenuated the expression of TGF‐β in CXCL6‐induced KCs compared with control cells exposed to CXCL6 + DMSO (*P* < 0.001). This result was verified by RT‐PCR analysis of the mRNA expression levels of TGF‐β on the same set of cells (Figure 2E). Western blot analysis confirmed that treatment with Afatinib attenuated the expression of phosphorylated EGFR and SCH attenuated the expression of CXCR1/2, and revealed that both SCH and Afatinib downregulated phospho‐ERK1/2 expression (Figure 2F). Taken together, these findings confirmed the role of the CXCR1/2‐EGFR pathway in CXCL6‐induced TGF‐β secretion in KCs.

### 3.3 CXCL6 can activate HSCs via KC conditioned medium

**Figure 3:**
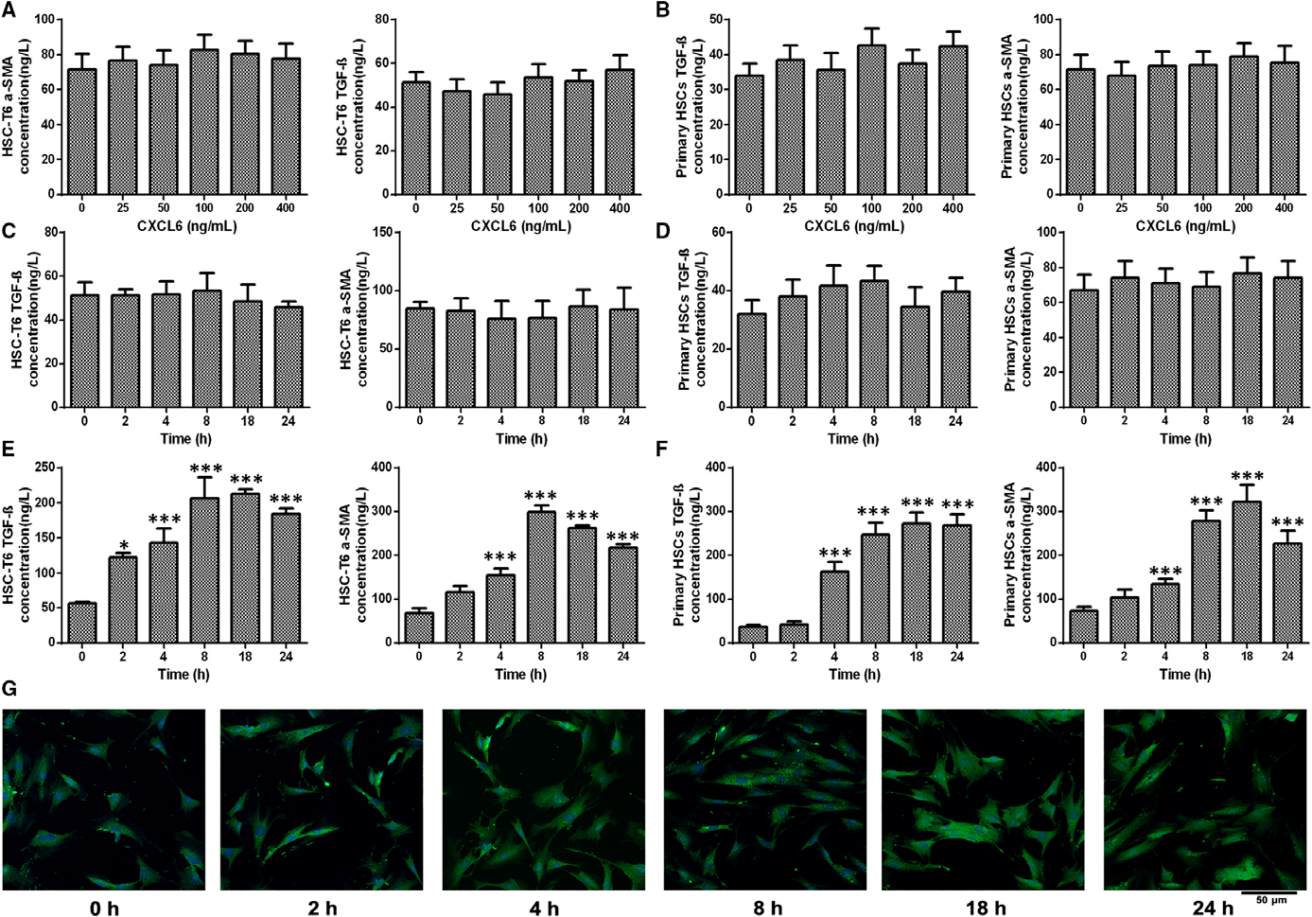
CXCL6 Activates Hepatic Stellate Cells via Kupffer Cell Conditioned Medium. A and B, α‐SMA and TGF‐β concentrations in hepatic stellate cell (HSC)‐T6 cells and primary rat HSCs incubated with CXCL6 for the indicated concentrations were detected by ELISA (n = 3). C and D, α‐SMA and TGF‐β concentrations in HSC‐T6 cells and primary rat HSCs incubated with CXCL6 (100 ng/mL) for the indicated times were detected by ELISA (n = 3). E and F, Kupffer cells (KCs) were treated with CXCL6 (100 ng/mL) for 18 h. KC conditioned medium was then transferred onto 3‐d HSCs and cultured for the indicated times. α‐SMA and TGF‐β concentrations in hepatic stellate cell (HSC)‐T6 cells and primary rat HSCs were detected by ELISA. G, α‐SMA in HSC‐T6 cells were cultured with KC conditioned medium was detected by immunofluorescence (n = 3). *P < 0.05, **P < 0.01, ***P < 0.001 vs 0 h group

The ability of CXCL6 to activate HSCs was tested by analysing the α‐SMA and TGF‐β concentrations in HSCs (HSC‐T6) incubated with CXCL6 over a 24 hour time‐course by ELISA (Figure 3A and B). The α‐SMA and TGF‐β concentrations remained unchanged in HSCs following incubation with CXCL6 indicating that CXCL6 was unable to activate HSCs directly. Next, it was hypothesized that KC conditioned medium containing CXCL6 may be capable of activating HSCs. To test this hypothesis, KCs were treated with CXCL6 for 18 hour, then KC conditioned medium was transferred onto 3‐day HSCs and cultured over a 24 hour time‐course (Figure 3C and D). ELISA revealed that the α‐SMA and TGF‐β concentrations were significantly upregulated, peaking at 8 hour for α‐SMA (*P* < 0.001 vs 0 hour group) and 18 hour for TGF‐β (*P* < 0.001 vs 0 hour group). Immunofluorescent staining of α‐SMA in HSCs cultured with KC conditioned medium confirmed α‐SMA upregulation, peaking at 8 hour (Figure 3E). These findings confirmed that exposure of HSCs to KC medium, conditioned by previous exposure to CXCL6, can activate these cells.

### 3.4 CXCL6 activates HSCs by stimulating TGF‐β and its downstream Smad3‐BRD4

**Figure 4:**
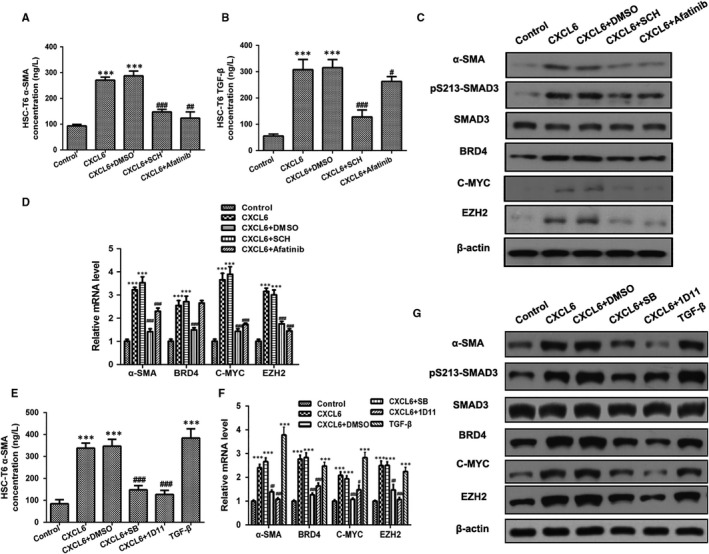
CXCL6 Activates Hepatic Stellate Cell (HSCs) by Stimulating TGF‐β and Downstream Smad3‐BRD4. A and B, Kupffer cells (KCs) were treated with CXCL6 (100 ng/mL) or phosphate‐buffered saline (PBS) with the addition of CXCR1/2 antagonist SCH527123 (10 μmol/L), EGFR antagonist Afatinib (10 μmol/L), or DMSO as a control for 18 h. KC conditioned medium was then transferred onto 3‐d HSCs and cultured for 8 h. α‐SMA and TGF‐β concentrations in HSC‐T6 cells were detected by ELISA. C and D, α‐SMA, SMAD3, BRD4, C‐MYC and EZH2 mRNA and protein levels in HSC‐T6 cells cultured with KC conditioned medium were detected by RT‐PCR and western blotting. E, KCs were treated with CXCL6 (100 ng/mL), TGF‐β (10 ng/mL) or PBS with the addition of TGF‐β receptor antagonist SB431542 (10 μmol/L), TGF‐β‐neutralizing antibody 1D11 (4 μg/mL) or DMSO as a control for 18 h. KC conditioned medium was then transferred onto 3‐d HSC‐T6 cells and cultured for 8 h. α‐SMA concentration in HSCs was detected by ELISA. F and G, Relative mRNA and protein levels in HSC‐T6 cells cultured with KC conditioned medium were detected by RT‐PCR and western blotting (n = 3). *P < 0.05, **P < 0.01, ***P < 0.001 vs control group, ^##^ *P* < 0.01, ^###^ *P* < 0.001 vs CXCL6 + DMSO group

To determine the pathway by which CXCL6 induces activation of HSCs, KCs were treated with CXCL6 with or without the addition of the CXCR1/2 antagonist SCH527123 or the EGFR antagonist Afatinib for 18 hour. Kupffer cells conditioned medium was then transferred onto 3‐day HSCs and cultured for 8 hour. ELISA on the HSCs revealed that the α‐SMA and TGF‐β concentrations were significantly downregulated in the inhibitor‐treated cells confirming that the CXCL6‐induced activation of HSCs was attenuated in the absence of CXCR1/2 and EGFR (Figure 4A and B). Furthermore, RT‐PCR and western blot analysis revealed that in HSCs cultured with KC conditioned medium, the mRNA and protein levels of α‐SMA, SMAD3, BRD4, C‐MYC, and EZH2 were significantly downregulated in the inhibitor‐treated cells compared with the CXCL6 + DMSO group confirming that the expression of these signalling components was attenuated in the absence of CXCR1/2 and EGFR in CXCL6‐activated HSCs (Figure 4C and D).

Next, to confirm the involvement of TGF‐β in the CXCL6‐mediated activation of HSCs, KCs were treated with CXCL6 or TGF‐β with or without the addition of the TGF‐β receptor antagonist SB431542 for 18 hour. Kupffer cells conditioned medium was then transferred onto 3‐day HSCs and cultured for 8 hour. Analysis of the α‐SMA concentration in HSCs by ELISA revealed significant activation of these cells in response to KC exposure to CXCL6 or TGF‐β (Figure 4E; *P* < 0.001 vs control group) that was attenuated on exposure to the TGF‐β receptor antagonist (Figure 4E; *P* < 0.001 vs CXCL6 + DMSO group). This same set of cells were then analyzed by RT‐PCR and western blotting and the mRNA and protein levels of α‐SMA, SMAD3, BRD4, C‐MYC and EZH2 were significantly downregulated in the inhibitor‐treated cells compared with the CXCL6 + DMSO group confirming that the expression of these signalling components is dependent on the induction of TGF‐β (Figure 4F and G). Taken together, these findings indicate that CXCL6 activates HSCs by stimulating TGF‐β and the downstream signalling components Smad3‐BRD4.

### 3.5 CXCL6 stimulates the Smad3‐BRD4 interaction and enhances the direct binding of BRD4 to the *C‐MYC* promoter and C‐MYC to the *EZH2* promoter, but not BRD4 to the *EZH2* promoter

**Figure 5:**
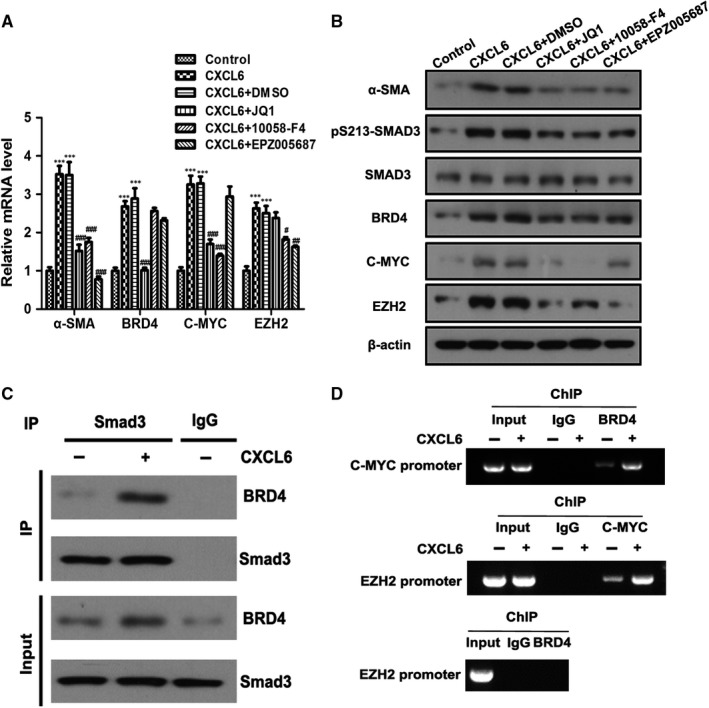
CXCL6 Stimulates Smad3‐BRD4 Interaction and Enhances the Direct Binding of BRD4 to the *C‐MYC* Promoter and CMYC to the *EZH2* Promoter. A and B, Kupffer cells (KCs) were treated with CXCL6 (100 ng/mL) or phosphate‐buffered saline (PBS) for 18 h. KC conditioned medium was then transferred onto 3‐d hepatic stellate cell (HSC)‐T6 cells with the addition of BRD4 antagonist JQ1 (1 μmol/L), C‐MYC antagonist 10058‐F4 (10 μmol/L) and EZH2 antagonist EPZ005687 (2 μmol/L) and cultured for 8 h. Relative mRNA and protein levels in HSC‐T6 cells cultured with KC conditioned medium were detected by RT‐PCR and western blotting. C, KCs were treated with CXCL6 (100 ng/mL) or PBS for 18 h. KC conditioned medium was then transferred onto 3‐d HSC‐T6 cells and cultured for 8 h. Whole cell extracts were immunoprecipitated with SMAD3 antibody or an equal amount of rabbit IgG and blotted with an BRD4 antibody. D, Chromatin immunoprecipitation was performed with the indicated antibodies (n = 3). *P < 0.05, **P < 0.01, ***P < 0.001 vs control group, ^##^ *P* < 0.01, ^###^ *P* < 0.001 vs CXCL6 + DMSO group

To investigate the signalling pathway in greater depth, KCs were treated with CXCL6 for 18 hour and KC conditioned medium was then transferred onto 3‐day HSCs along with the BRD4 antagonist JQ1, the C‐MYC antagonist 10058‐F4 or the EZH2 antagonist EPZ005687, and the cells were cultured for 8 hour. RT‐PCR and western blotting analysis revealed that all three inhibitors attenuated α‐SMA expression, the BRD4 antagonist JQ1 also attenuated BRD4 and C‐MYC expression but not EZH2 expression, the C‐MYC antagonist 10058‐F4 attenuated C‐MYC and EZH2 expression but not BRD4, and the EZH2 antagonist EPZ005687 attenuated EZH2 expression but not BRD4 or C‐MYC expression (Figure 5A and B). The expression of phosphorylated SMAD3 was downregulated following treatment with all three of the inhibitors (Figure 5B).

Next, KCs were treated with CXCL6 for 18 hour, then the KC conditioned medium was transferred onto 3‐day HSCs and cultured for 8 hour. Whole cell extracts were immunoprecipitated with SMAD3 antibody or an equal amount of rabbit IgG and blotted with an BRD4 antibody (Figure 5C). Exposure to CXCL6 led to the co‐immunoprecipitation of the SMAD3 and BRD4 antibodies indicating that CXCL6 stimulates the interaction between Smad3 and BRD4. Finally, a ChIP assay was performed on the same set of cells using the SMAD3 and BRD4 antibodies (Figure 5D). The results indicated that CXCL6 stimulates the direct binding of BRD4 to the C‐MYC promoter and C‐MYC to the EZH2 promoter, but not BRD4 to the EZH2 promoter.

### 3.6 Role of CXCL6 verified in a mouse model of chronic liver injury and fibrosis

**Figure 6:**
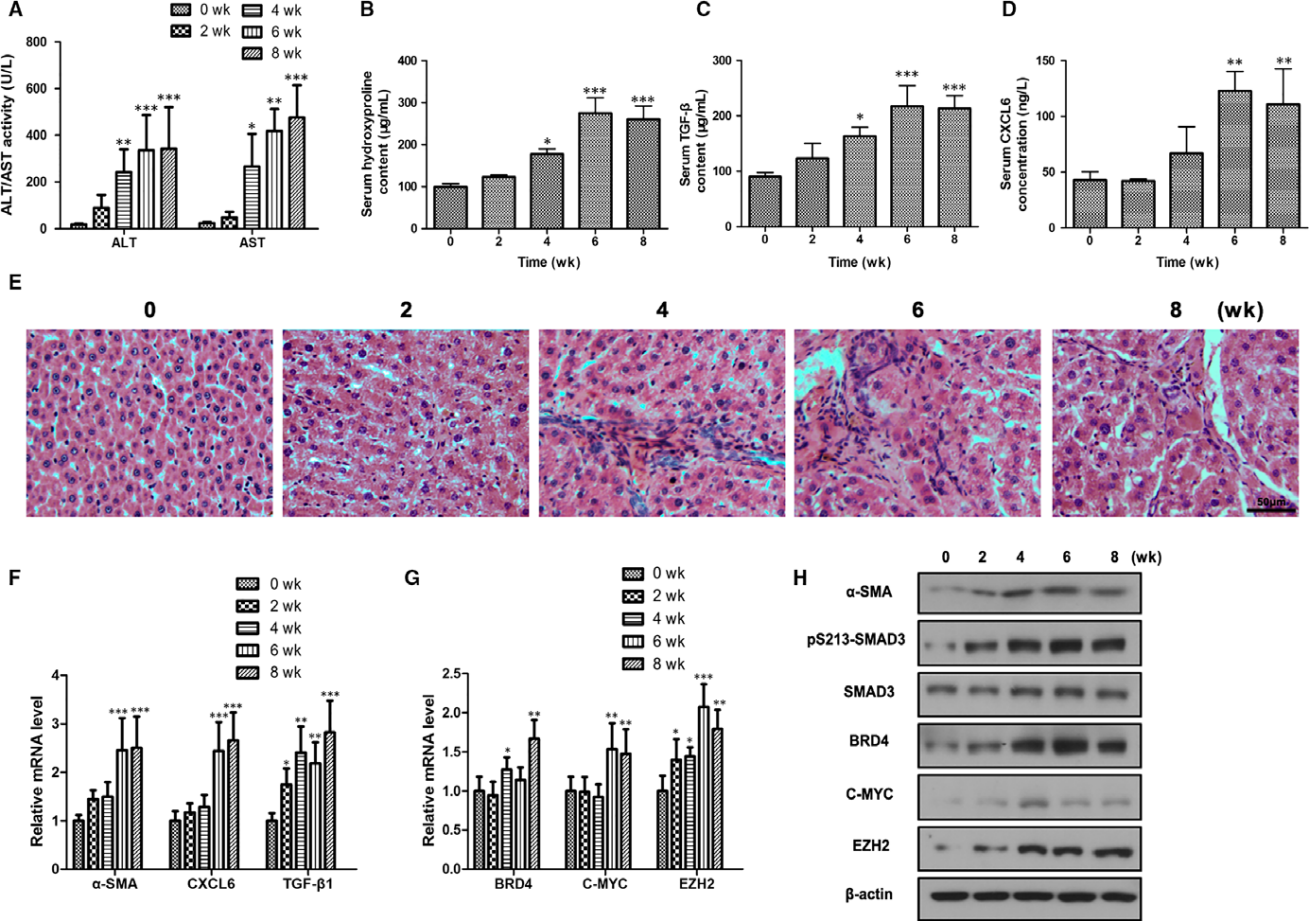
CXCL6 is Highly Expressed in a Long‐Term CCl_4_‐Induced Liver Fibrosis Mouse Model. A, Serum ALT/AST activity was detected after mice had been treated with CCl_4_ (200 or 300 mg/kg) for 0, 2, 4, 6 or 8 wk. B‐D, Serum hydroxyproline, TGF‐β and CXCL6 levels were determined. E, Representative images of H&E stained liver sections (×400 magnification). F‐H, Relative mRNA and protein levels in the mouse liver tissues were detected by RT‐PCR and western blotting (n = 10). *P < 0.05, **P < 0.01, ***P < 0.001 vs 0 wk group

A mouse model of chronic liver fibrosis was generated by injecting mice regularly with CCl_4_ over several weeks (0, 2, 4, 6 and 8 weeks) to induce high expression of CXCL6 and increasing severity of liver pathology. Experiments were then performed to verify the role of CXCL6 in liver fibrosis in vivo. A steady increase in serum ALT/AST activity accompanied the progression of liver fibrosis in the mice (0‐8 weeks of treatment, *P* < 0.001 at 8 weeks vs the 0 weeks group), indicating deterioration of liver health (Figure 6A). Similarly, a steady increase in serum hydroxyproline, TGF‐β, CXCL6 levels and steatohepatitis of liver sections detected by H&E stained was observed, peaking at 6 weeks of treatment (*P* < 0.001, *P* < 0.001 and *P* < 0.01 vs the 0 weeks group, respectively) and slightly decreasing at 8 weeks (Figure 6B‐F).

The mRNA and protein levels of α‐SMA, SMAD3, CXCL6, TGF‐β, BRD4, C‐MYC and EZH2 were then detected in mice liver tissues by RT‐PCR and western blotting (Figure 6F‐H). The expression of all of these signalling components was significantly upregulated along with the progression of liver fibrosis in the mice, in each case peaking at either 6 or 8 weeks of treatment. These findings verified the role of CXCL6 in promoting the secretion of TGF‐β and activating HSCs via the SMAD2/BRD4/C‐MYC/EZH2 pathway during liver fibrosis in vivo.

## 4 DISCUSSION

The targeting of signalling pathways that perpetuate myofibroblast activation is a promising therapeutic strategy to combat chronic fibrosis. Fibrosis contributes to the pathogenesis of a number of chronic disorders affecting the liver, kidney, lungs and heart, which collectively account for a substantial proportion of disease‐related mortalities in developed countries.^28^ Despite recent FDA approval of the first antifibrotic therapies, alternative treatments are needed. To achieve this, an in‐depth understanding of the complexities of the activation and transdifferentiation of macrophages into a fibrogenic phenotype is required.

CXC chemokines have previously been reported to play a role in the pathogenesis of various fibrotic diseases. In humans, there are seven CXC chemokines containing the ELR motif (CXCL1, 2, 3, 5, 6, 7, 8) that interact with the G protein‐coupled receptors CXCR1 and/or CXCR2. Many of these proteins have been characterized as neutrophil chemoattractants,^29^ for example, CXCL5‐deficient mice exhibited decreased recruitment of macrophages into the lungs in a model of cigarette‐smoke inflammation.^30^ Furthermore, CXCL6 has been shown to play a role in neutrophil recruitment leading to tissue damage and prolonged inflammatory responses.^22^ CXCL6 was thereby proposed to contribute to fibrosis.

In this study, patients with various stages of liver fibrosis were analysed and elevated expression of CXCL6 was detected in the sera and liver tissue, with higher expression levels correlating with more advanced stages of the disease. However, our findings indicated that CXCL6 was unable to activate resident HSCs directly. Therefore, we tested whether KCs, which also reside in the liver and may potentially be recruited by CXCL6, may be responsive to this chemokine. Exposure of KCs to CXCL6 was found to increase secretion of TGF‐β, accompanied by increases in expression of p‐EGFR, pERK1/2 and CXCR1/2. This confirmed the role of the CXCR1/2‐EGFR pathway in CXCL6‐induced TGF‐β secretion in KCs. The next step was to analyse whether the media from KCs cultured with CXCL6 could activate HSCs. This was confirmed indicating that KCs residing alongside HSCs in the microenvironment of the liver may modulate HSC behaviour via the secretion of profibrogenic TGF‐β. Although HSCs, which constitute around 10% of resident liver cells, are phenotypically non‐proliferative and quiescent in healthy livers, upon activation these cells transdifferentiate into proliferative, inflammatory, fibrogenic myofibroblasts. This transformation, triggered by signalling pathways such as those involving PDGF and TGF‐β, drives liver fibrosis in response to tissue injury. Using specific inhibitors, we demonstrated that TGF‐β‐induced HSC activation involved the downstream signalling components Smad3‐BRD4. Again, using specific antagonists, we depicted that BRD4 could interact with the *C‐MYC* promoter to upregulate its expression, and subsequently enhance the direct binding of C‐MYC to the *EZH2* promoter. This signalling pathway would lead to the promotion of profibrogenic gene expression, triggering the differentiation of HSCs into a proliferative, fibrogenic form. Our findings were verified in vivo in a mouse model of chronic liver fibrosis.

The elevated expression of CXCL6 in the sera and liver tissue of liver fibrosis patients identified in this study suggests that this chemokine could be an effective marker of liver fibrosis. Synovial macrophages are the major source of most CXC chemokines. However, the specific source of CXCL6 in fibrotic livers remains unclear. KCs are specialized macrophages that reside in the liver and secrete a variety of proinflammatory and profibrogenic factors such as cytokines, chemokines, prostaglandins, leukotrienes and complement factors in response to various stimuli.^31^ Chemokines reported to be released by KCs include CXCL1, CXCL2 and CXCL8, which attract neutrophils,^32^ CCL1, CCL2, CCL25 and CX3CL1, which promote the infiltration of bone marrow−derived monocytes,^33^, ^34^, ^35^, ^36^ and CXCL16, which attract natural killer T cells.^37^ We therefore speculate that activated KCs may be the source of CXCL6 in fibrotic livers and that the CXCL6 secreted by these macrophages, in turn, promotes the release of TGF‐β by KCs via the CXCR1/2‐EGFR pathway. Kupffer cells reside alongside HSCs in the liver, therefore the secreted TGF‐β has profibrogenic effects on neighbouring HSCs.

As stated above, our findings revealed that CXCL6 activated the CXCR1/2‐EGFR pathway in KCs. However, interestingly, CXCL6 did not activate HSCs via this same pathway, despite the fact that CXCR1/2‐EGFR is also expressed in HSCs. In HSCs, CXCL6 must induce activation via a distinct pathway. This may be investigated further in subsequent studies.

One of the limitations of our study was that we only investigated the secretion of TGF‐β in KCs in response to CXCL6. TGF‐β is considered the predominant cytokine to trigger HSC activation and fibrogenic transdifferentiation; however, it is possible that CXCL6 may alter the secretion of other fibrosis‐related cytokines in KCs. This should be addressed in future studies. Another limitation was that we did not genetically knock down the expression of liver CXCL6 to confirm its role in promoting the secretion of TGF‐β and activating HSCs in vivo. Future studies analysing the effects of knockdown mutants in the animal model would verify our findings.

In conclusion, our findings reveal the role of CXCL6 in promoting the release of TGF‐β by KCs. TGF‐β is a potent inducer of HSC activation via a signalling cascade that involves the SMAD2 protein and the BRD4/C‐MYC/EZH2 axis. HSC activation and the resulting transdifferentiation into fibrogenic myofibroblasts play a pivotal role in the pathogenesis of liver fibrosis. Our findings, therefore, provide important insight into the complex mechanisms of HSC activation that contribute to liver fibrosis.

## CONFLICTS OF INTEREST

The authors declare that they have no conflict of interests to disclose.
